# Antelope space‐use and behavior indicate multilevel responses to varying anthropogenic influences in a highly human‐dominated landscape

**DOI:** 10.1002/ece3.9372

**Published:** 2022-10-01

**Authors:** Rohit Raj Jha, Kavita Isvaran

**Affiliations:** ^1^ Post‐graduate Program in Wildlife Biology and Conservation National Centre for Biological Sciences Bangalore India; ^2^ Centre for Ecological Studies Lalitpur Nepal; ^3^ Centre for Ecological Sciences Indian Institute of Science Bangalore India

**Keywords:** behavior, conservation, generalist grazer, habitat use, human‐dominated landscape

## Abstract

A primary means of conserving a species or a habitat in a human‐dominated landscape is through promoting coexistence with humans while minimizing conflict. For this, we should understand how wildlife is impacted by direct and indirect human activities. Such information is rare in areas with high human densities. To investigate how animals respond to altered ecological conditions in human‐dominated landscapes, we focused on a wild herbivore of conservation concern in the Krishnasaar Conservation Area (KrCA) in Nepal. Here, blackbuck *Antilope cervicapra*, a generalist grazer, lives in refugia located with a growing human population. We studied the impacts of humans on habitat use and behavior of blackbuck. We laid 250 × 250 m grid cells in the entire KrCA and carried out indirect sign surveys with three replications for habitat use assessment. We observed herds of blackbuck for 89 h in different habitat types using scan sampling methods. Our habitat‐use survey showed that habitats under intensive human use were hardly used by blackbuck, even when high‐quality forage was available. Habitat openness was the major predictor of habitat use inside the core area, where levels of human activities were low. We also found a positive correlation between habitat use by blackbuck and livestock. Blackbuck were substantially more vigilant when they were in forest than in grassland, again indicating an influence of risk. Overall, blackbuck appear to be sensitive to the risk associated with both natural and anthropogenic factors. Our findings have direct implications for managing human–wildlife interactions in this landscape, specifically regarding strategies for livestock grazing in habitats highly used by blackbuck and concerning predictions of how changing land use will impact the long‐term persistence of blackbuck. Our work suggests that wild herbivores may be able to persist in landscapes with high human densities so long as there are refuges where human activities are relatively low.

## Graphical abstract

To investigate how animals respond to altered ecological conditions in human‐dominated landscapes, we focussed on a wild herbivore of conservation concern in Nepal. Our habitat‐use survey showed that habitats under intensive human use were hardly used by blackbuck and they were more vigilant when in smaller herd and in closed habitat. Our work suggests that wild herbivores may be able to persist in landscapes with high human densities so long as there are refuges where levels of human activity are relatively low.

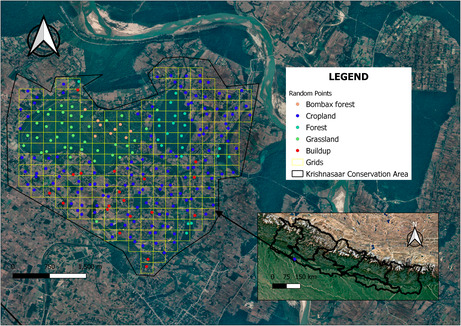

## 1 INTRODUCTION

Anthropogenic influences are rapidly pervading habitats worldwide (Maier et al., 2005; Schlaepfer et al., 2018; Stewart et al., 2010). In South Asian nations including Nepal, a large part of the populations or range of many species, including large and charismatic species, occurs within highly human‐dominated landscapes. For example, the blue bull *Boselaphus tragocamelus* (Khanal et al., 2018; Meena et al., 2014), blackbuck *Antilope cervicapra* (Jhala & Isvaran, 2016), Indian wolf *Canis lupus pallipes* (Sharma et al., 2019) and the critically endangered Great Indian bustard *Ardeotis nigriceps* (Dutta et al., 2011) are now primarily found among dense human populations in most of their distribution range. A key step toward devising conservation strategies for such species is to understand how they respond to the modifications to their habitats by anthropogenic drivers.

The major impact of human presence in a habitat is its modification. Human‐induced modifications of landscapes can have both positive and negative impacts on wildlife. Adverse impacts can occur through habitat loss, fragmentation of already isolated patches, and a continuous increase in direct and indirect human presence (Maier et al., 2005; Stewart et al., 2010). Animals are thought to respond to these modified conditions in different ways. Several studies report shifts in the behavior of animals in human‐dominated landscapes (Ditchkoff et al., 2006; Riley et al., 2003; Tigas et al., 2002; Valeix et al., 2012). For example, moose (*Alces alces*) browsing sites depended both on the availability of browse and distance from the road indicating that moose trade‐off foraging with maintaining distance from the road. Moose even maintained different distances from roads that differed in the intensity of human use (Eldegard et al., 2012). Many of these behavioral shifts are proposed to result from how the ecology of fear operates (Clinchy et al., 2016). Animals may perceive humans as “super predators” and may even show greater antipredator responses toward humans than toward natural predators (Bonnot et al., 2020; Zbyryt et al., 2018).

Given that human activities are known to alter ecological conditions for animal species, understanding how animals respond to these altered conditions is essential for effective conservation planning. Approaches to manage and conserve populations in such human‐modified landscapes are likely to differ from those used for animals primarily living inside protected areas. However, we continue to lack key information on behavioral responses of wild animals in landscapes with high human density (e.g., multiuse human‐dominated landscapes in the global south with densities such as ~200 humans/km^2^) (Krishna et al., 2016). Previous work on behavioral responses to anthropogenic factors has primarily been carried out in areas with comparatively low human density (e.g., *Bison bonasus* response to human disturbance within a protected forest with ~30 humans/km^2^; Haidt et al., 2018) and other similar studies (Frey et al., 2020; Mendes et al., 2020).

To address how animals respond to altered ecological conditions in human‐dominated landscapes, we focused on the behavioral responses of a habitat generalist, blackbuck *Antilope cervicapra*, sharing a landscape with humans in Nepal. This species and study site are well‐suited to a study of the impacts of anthropogenic factors on the ecology and behavior of a relatively large species like blackbuck. The study site, Krishnasaar Conservation Area (KrCA), is of conservation importance as it is the only landscape that hosts a wild population of blackbuck in the country. The study species are of high conservation priority in Nepal as there are only around 250 individuals in the wild in Nepal. The species is locally threatened and is one of 27 mammal species that is legally protected by the Nepal Government under the National Park and Wildlife Conservation Act, 1973 (KrCA, 2017).

How should ungulates like blackbuck respond to the modified ecological conditions of human‐dominated landscapes? Ungulate habitat use and behavior are sensitive to multiple risk and resource factors (Anderson et al., 2010). In areas where human presence is low, they are known to pay attention to habitat structure that affects their interaction with wild predators. Herbivores, in general, experience a “landscape of fear” (Anderson et al., 2010; Verdolin, 2006). That is, they trade off predation risk against foraging benefits and are thought to avoid foraging in areas with high‐quality forage if these areas carry a sufficiently high level of predation risk. Such predation risk is often assessed through indirect cues, such as habitat structure, rather than direct cues such as predator presence (van der Merwe & Brown, 2008). The habitat use of ungulates is also affected by the quality and quantity of resources (Belovsky, 1981). For example, roe deer (*Capreolus capreolus*) and fallow deer (*Dama dama*) were shown to prefer habitats with small shrubs over those with large trees, and this difference in habitat use was explained well by differences in the distribution of resources (Heinze et al., 2011). The direct and indirect presence of humans is likely to affect both the “landscape of fear” and resource factors experienced by ungulates (Bonnot et al., 2013). For example, a study that examined the antipredator behavior of multiple ungulate species in relation to human presence found that giraffe (*Giraffa camelopardalis*) and zebra (*Equus quagga*) showed a stronger flight response when they were closer to human settlements (Yamashita et al., 2018). Mule deer experienced a net loss in food as a result of their space‐use responses to human disturbance factors (Dwinnell et al., 2019).

**FIGURE 1:**
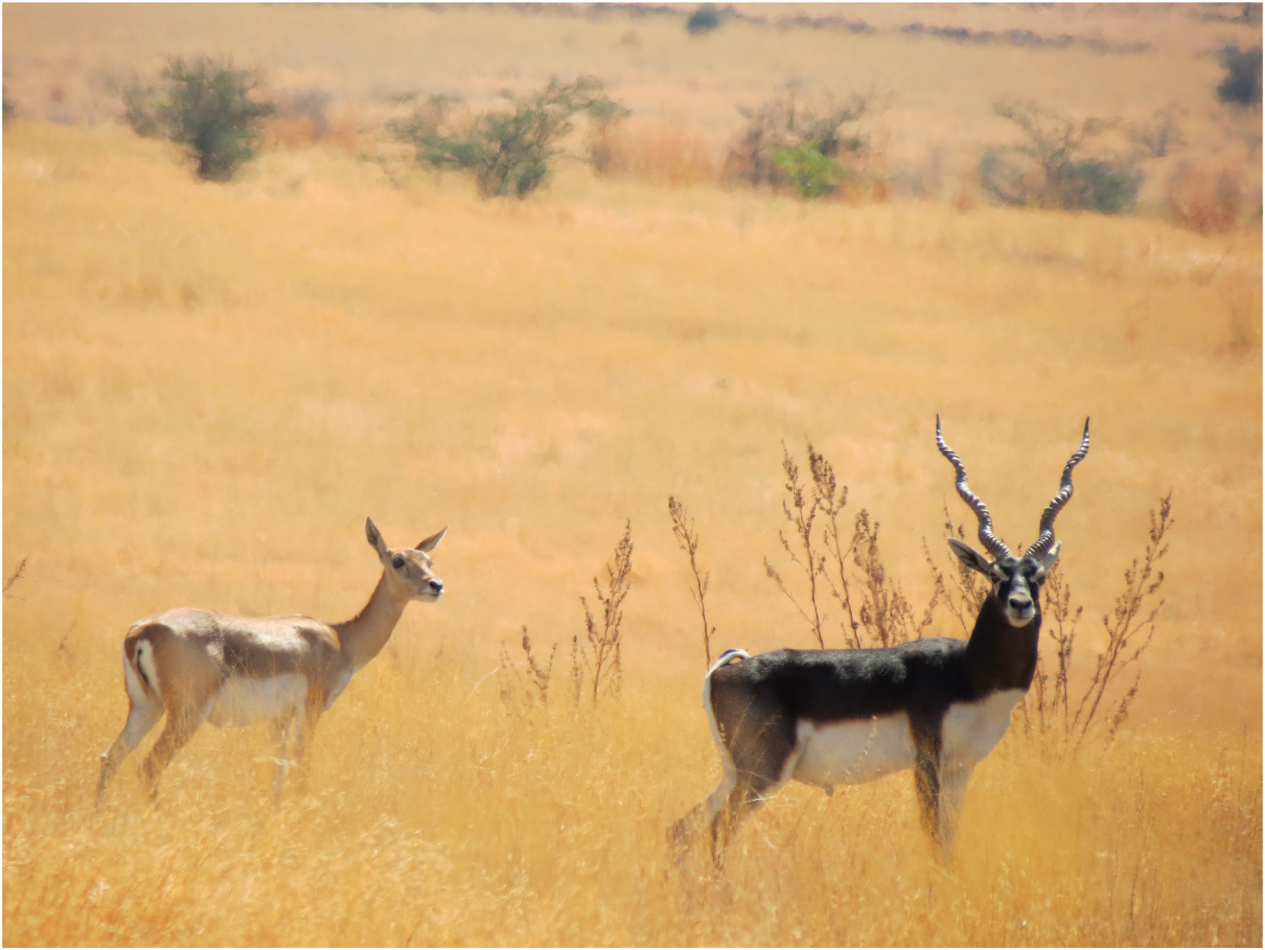
A male and a female blackbuck (*Antelope cervicapra*) at Great Indian Bustard Sanctuary Nannaj, India (photo credit: Sarang Mhamane).

Blackbuck are well‐suited for a study of wild herbivore responses in human‐dominated landscapes. They are open‐habitat, group‐living, generalist grazers found in habitats ranging from semi‐dry grasslands to open forests (Figure 1; Isvaran, 2005; Ranjithsinh, 1989). The behavior, nutritional ecology, breeding biology, and demography of this species suggest that they are highly specialized to open, short grass, semi‐arid habitats (Jhala & Isvaran, 2016). These animals use group‐living, early detection, and flight when faced with an approaching risk or predator (Mungall, 1978). Although blackbuck occur in multiuse landscapes with high human densities in most of their range, they appear to be risk averse and tend to avoid high levels of human activity (Krishna et al., 2016). Blackbuck face anthropogenic factors commonly experienced by wild herbivores in areas with high human density. First, they share foraging areas with livestock, a feature that is common in human‐dominated dry landscapes (KrCA, 2017). The impact of livestock on wild herbivores is still not well resolved. Khanal and Chalise (2011) suggests that sharing common foraging space by livestock and blackbuck is not beneficial for the latter as livestock remove a large quantity of resources that could otherwise be used by blackbuck. Second, blackbuck are frequently reported to feed on crops (Das et al., 2018; Jhala, 1993). Such crop use is one of the main sources of human‐wildlife conflict associated with large wild herbivores (Bhatta, 2008; Meena & Jaipal, 2020). While crop fields provide high‐quality forage, they are also associated with risks due to direct and indirect human presence (e.g., related to guarding crops). Therefore, there are several factors associated with risks in human‐dominated landscapes, even in the absence of hunting. However, we still lack a comprehensive understanding of how wild species cope with these risks through their use of different habitats and through their social behavior.

We explored the impact of natural and anthropogenic factors on habitat‐use and behavior patterns by blackbuck in a human‐dominated landscape. We first focused on habitat use and asked (1) how do blackbuck vary their use of habitats that differ in the level of human activity and in natural ecological conditions like habitat structure and forage abundance? For an open‐habitat grazer like blackbuck, we hypothesized that resource and risk factors will jointly affect habitat‐use patterns by blackbuck in human‐dominated landscapes. We expected a natural risk factor, namely the degree of openness of the habitat (with closed perceived as risky and open as safe) to affect blackbuck habitat use. We also expected anthropogenic risk factors, specifically, the distance from the edge of protected areas, and livestock densities, to affect habitat use. In addition, we also expected an influence of natural (grass abundance) and anthropogenic (crop availability) resources on blackbuck habitat use.

Second, to understand how animals might respond to anthropogenic conditions through changes in behavior, we asked (2) how does blackbuck activity vary across the different habitat types that they visit? Antelope like blackbuck use vigilance and group formation to reduce the risk of predation. Specifically, in natural ecological conditions, larger groups may reduce individual risk and investment in vigilance (Isvaran, 2007). Here, we hypothesized that (3) animals should modify their vigilance in response to the risk associated with habitat types. We predicted that animals would be more vigilant in habitat patches that are perceived as high risk, specifically, habitats, which are more closed and with higher human activity. (4) We also expected that animals in smaller herds would be more alert than those in larger herds as predicted by the theory of living in groups (Krause et al., 2002).

## 2 MATERIALS AND METHOD

### 2.1 Study area

**FIGURE 2:**
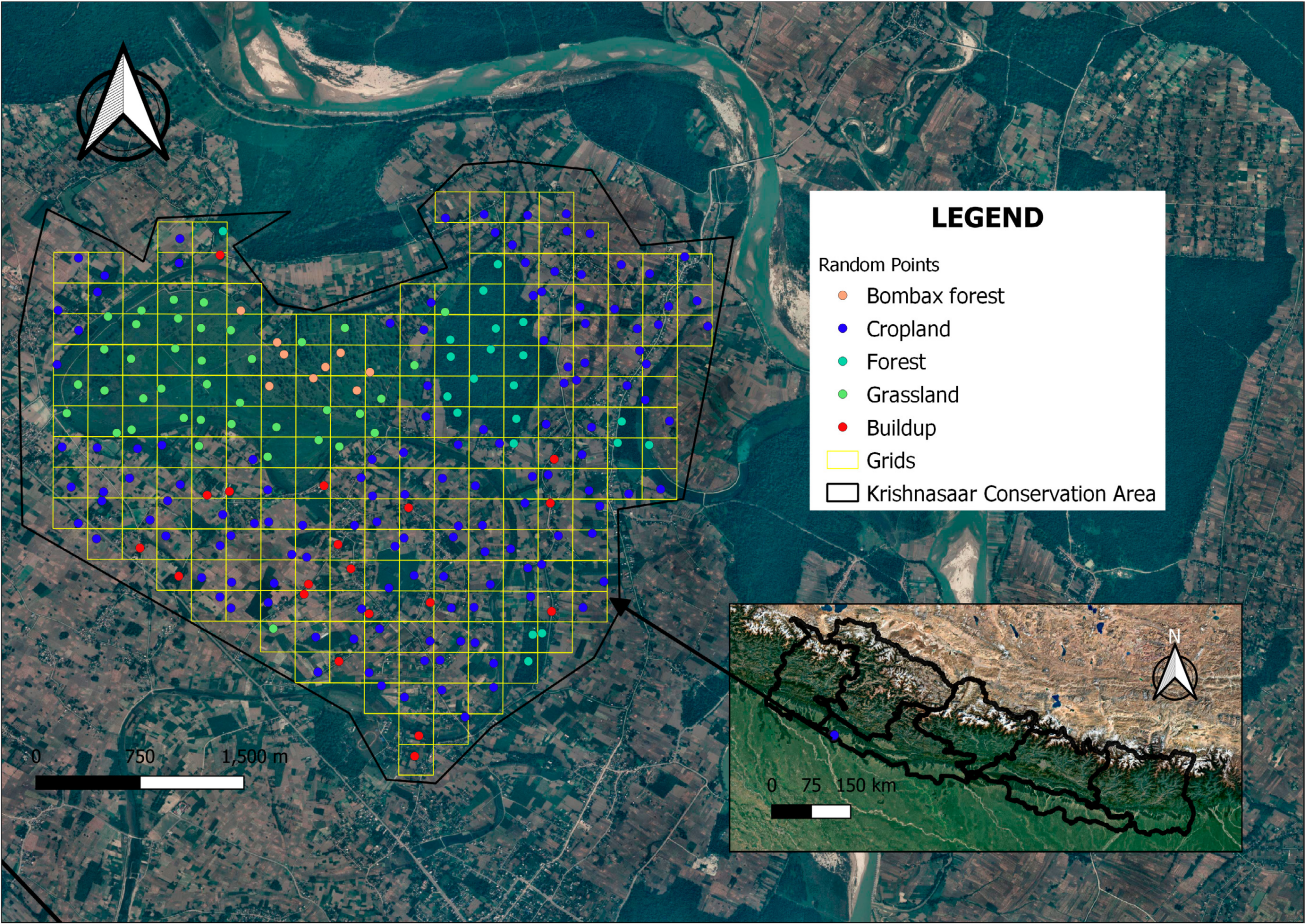
Satellite imagery of the study site showing different habitat types.

We studied blackbuck in Krishnasaar Conservation Area (KrCA), which is situated in Gulariya municipality of western lowland Terai of Nepal (Figure 2). It lies between 28°7′ and 28°39′N latitude and 81°3′ and 81°4′E longitude. KrCA, measuring 16.95 sq. km. in area, is divided into two areas that experience different management regimes: the Core Area (CA) of 5.27 sq. km and the Community Development Zone (CDZ) of 11.68 sq. km. KrCA represents a conservation area with high human and livestock densities. The CA has around 150 households and about 500 cattle. The CDZ consists of built‐up areas and crop fields, with no grassland or forest patches. Similarly, the CDZ has 1669 households with a total population of 8789. The total number of livestock recorded from those households was 2384 (KrCA, 2017).

The major habitat types in the core of KrCA are grassland, open forest with *Bombax ceiba* as the major tree species, crop fields, and dense forest with *Lantana camara* as dense undergrowth. The main grass species in the core of KrCA are dubo (*Cynodon dactylon*), mothe (*Cyperus rotundus*), jwano grass (*Fimbrisrysis dichotoma*), and siru (*Imperata cylindrical)*. Blackbuck are the only mammalian wild herbivore in the landscape with blue bull (*Boselaphus tragocamelus*) as an occasional visitor. Livestock, mainly cattle, buffalo, and goats, are the other herbivore species that usually graze on the fringes of the core area of KrCA.

Blackbuck appear to face high predation pressure in the landscape. Body parts of blackbuck were recovered on six occasions during the study period, all of which were attributed to attacks by leopard (*Panthera pardus*) (Lead author's personal observations). Other than leopard, the core area is also home to predators like golden jackal (*Canis aureus*), hyena (*Hyaena hyaena*), and Bengal foxes (*Vulpes bengalensis*). Information from KrCA officials indicated that leopards are major predators in the landscape and are active throughout the year. Golden jackal, hyena, and foxes mainly target fawns as their prey and are active during the fawning season (March–April). Increasing incidences of chases and attacks by stray dogs on blackbuck were also reported.

### 2.2 Method

#### 2.2.1 Habitat‐use pattern

The study area was divided into 224–250 m × 250 m grids using QGIS. The sizes of grids were decided based on the daily movement range of blackbuck, which can range from 1.5 to 5.7 km (Jhala & Isvaran, 2016). This grid size allows us to examine how animals use different habitats available to them. A 20 m buffer was set around the perimeter of each grid and a sampling point was randomly placed within the grid while avoiding the buffer area. This would ensure a distance of at least 40 m between the random points and the independence of sampling points. The main habitat types in these grids were grassland, forest, croplands, and built‐up areas. To collect blackbuck sign data as a proxy of habitat use (Krishna et al., 2016), we laid out a strip transect of dimension 20 × 4 m at the chosen sampling point in each of the sub‐grids. The strip was divided into five segments of four meters each, and each segment was scored for the presence of indirect signs of blackbuck (pellet groups).

The primary risk factor we measured for each sampling point (strip transect) was a measure of habitat openness. We counted the number of woody plants over 1 m in height in a 10 m radius plot, centered on the starting point at each transect. We used this method and chose 1 m as the cut‐off for woody plant height because multiple previous studies have used a similar approach (Isvaran, 2007; Krishna et al., 2016). One of the reasons for choosing 1 m as the cut‐off is that given the approximate height of blackbuck, their line of sight would roughly be 1 m.

The second risk factor we considered was the intensity of livestock use in the area. For this, the indirect livestock signs for each strip transect were summarized as the sum of dung piles and hoof marks of cattle present in the strip. We also calculated the distance of each sample point from the centroid of the core area. This distance measure was considered as a third risk factor because the periphery had settlements, crop fields, or forest patches (potential anthropogenic and natural risk factors for blackbuck). Each strip transect was sampled three times over 3 months (December 2019 to February 2020). This yielded 213 data points from CA and 459 data points from CDZ.

At each strip transect of 20 m, we measured the height and cover percentage of grasses/herbs in two 1 m × 1 m plots located at 0 and 20 m points along the length of the strip transect. Grass/herb height at each 1 m × 1 m plot was measured using a measuring tape whereas grass/herb cover percentage was visually estimated. These data were used to calculate a proxy of resource abundance for blackbuck, which is primarily a grazer. We averaged the two values to get the average resource abundance at each strip transect. Grass height and cover have been used to index resource availability by previous studies (Isvaran, 2007; Maher, 2000).

#### 2.2.2 Behavior assessment

We used scan sampling methods for groups to record the behavior of blackbuck in the different habitat types and zones (Altmann, 1974). In this method of recording behavior, a group of animals is selected for observation. The group was defined by including all individuals that are within 50 m of another individual (Isvaran, 2005; Lingle, 2001). We made our observations from raised structures (view‐towers) or stood at least 60–80 m from the herd. At the start of the observation, we recorded the total number of individuals in the group and their age and sex composition. Age composition was possible only in the case of males where the size of the horn and curl in the horn were used to distinguish males as immature or adult (Isvaran, 2005). We categorized individuals as fawn (sex not separated), immature male (from horns visible up to three curls in the horn), female (size larger than fawns but no visible horn), and adult male (more than three curls in the horn). All observations were conducted between 0505 h and 1815 h in December 2019, and in January and February 2020.

An observation session of a group lasted for 1 h. Every 10 min from the start of the session (zero minute) to the 60th minute, we scanned the group and recorded the behavior of all individuals at that instant (using a pair of binoculars, Nikon Action EX 8x40 8.2^0^)^.^ This gave us seven scans for each hour of observation. During each scan, the activities shown by each individual of different age classes and sexes were noted. The activities we considered were forage, lie, stand, move, chase, and fight (Meena & Chourasia, 2017) (Definitions in Appendix S1). We also noted the type of habitat (grassland, forest, cropland), the broad zone (CA or CDZ) the group was located, and weather condition at the time of observation.

#### 2.2.3 Vigilance behavior

We defined a typical vigilance behavior as when an animal raises its head and scans its surrounding (Beauchamp, 2015). We sampled adult females only to reduce variation in the data that would arise from age‐ and sex‐related behavioral responses (Isvaran, 2007). To measure vigilance behavior, we sampled a female from a group continuously for 1 min (continuous focal animal sampling, Altmann, 1974). We sampled up to three females from a group. We selected the first female at random, and the subsequent females systematically to ensure that they were at least 20 m away from the first female. If another herd was selected for observation on the same day, it was always located at some distance from the first. During each observation, the number of times the female raised her head up and looked around was noted. The group size, time of the day, habitat type, zone, and weather were also recorded for each vigilance observation.

### 2.3 Analysis

#### 2.3.1 Habitat‐use pattern

For each 20 × 4 m strip transect, the possible score for habitat use ranged from 0 (no pellet groups of blackbuck) to 5 (pellet groups present in all 5 segments of the transect).

##### Modeling landscape level habitat use

As blackbuck presence data outside the core area (CA) were scarce (only 2 out of 459 trials), it was not meaningful to examine the predictors of habitat use outside the CA. Therefore, we excluded all the observations outside the core and used 213 data points obtained from three rounds of sampling inside the CA for modeling habitat use. Since our response variable, the indirect signs score for habitat use, was in the form of count data, we used Generalized Linear Mixed Models (GLMMs) with a Poisson error structure. Number of woody plants over 1 m, resource abundance, livestock signs, and distance from the centroid of the CA were used as predictor variables. We included two interactions. We modeled the interaction between the number of woody plants over 1 m and resource abundance. We similarly included the interaction between distance from the core centroid and resource abundance. The identity of sampling points was treated as a random effect because sampling points were repeatedly measured. The four predictor variables were checked for multicollinearity through pair‐wise correlations.

All the analyses were run in the software R 4.0.2 (R Core Team, 2013) using the package “glmmTMB” (Brooks et al., 2017). Our statistical inferences were based on the model selection framework using an information‐theoretic approach (Johnson & Omland, 2004). The information‐theoretic approach examines the strength of evidence for different candidate models and identifies one or more models that best fit the data. This approach permits multimodel inference through model averaging of parameters when multiple models provide a similar fit to the data (Barton, 2018). Based on our hypotheses and knowledge of blackbuck ecology and behavior, we framed an a priori candidate set of 24 models, including the null and the global models, each representing a different ecological hypothesis (Appendix S2). These models included either single or additive effects of two or more covariates. We ranked different models using Akaike Information Criterion (AIC) and models with ΔAIC of <2 from the best fit models were considered statistically indistinguishable (Burnham & Anderson, 2002). We used the estimated β‐coefficients and their 95% confidence limits to assess the strength of each term in the model. Since no single model appeared to best fit the data, we used multimodel averaging to estimate the parameters using the R package “MuMIn” (Barton, 2018).

#### 2.3.2 Behavior assessment

The data obtained through instantaneous scan sampling were used to calculate the proportion of time animals devote to each activity. Each observation session of a group had seven scans. From these data, the proportion of time spent in a particular activity (e.g., foraging) by an average individual in the group was calculated as the sum of all individuals showing that activity across the 7 scans divided by the sum of all individuals sampled across all seven scans. Thus, for each observation session of a group, the proportion of time spent in foraging, standing, moving, laying, chasing, and fighting was calculated. For each session, the mean group size across the 7 scans was calculated.

##### Modeling foraging and moving behavior

We analyzed variation among groups in the proportion of time spent foraging using beta regression models. Beta regression is used when the response variables are probabilities in themselves, i.e., the value of the response variable ranges between zero and one (Cribari‐Neto, 2010). Since the response variable had some zeros and ones, violating the assumptions of beta regression, we transformed the proportion data as recommended (Smithson & Verkuilen, 2006). We did not take the approach of adding an offset since some of the values were exactly one.

Similarly, we used beta regressions to analyze inter‐group variation in the proportion of time spent moving. The response variable had many zeros and the highest values were well below one. Thus, we added an offset to each dependent value to meet the assumption (variable must range from >0 to <1) for fitting beta regressions.

The predictor variables for modeling both foraging and moving behavior were mean group size; habitat type (grassland, *Bombax* forest); location (core and settlement); group type (female only, male only, mixed); weather (no sun, partial sun, sunny); and time of the day (day, evening, morning). The analyses were run using the package betareg (Cribari‐Neto, 2010). We framed an a priori candidate set of 26 models for both foraging (Appendix S3) and moving (Appendix S4) behavior including both the null and the global models, each representing a different ecological hypothesis.

##### Modeling vigilance behavior

To model vigilance behavior (number of times a female raised her head up in a minute), we used GLMMs with Poisson error distribution. Each female observed constituted a data point.

The predictor variables were mean group size, habitat type (grassland, *Bombax* forest), location (core, settlement); group type (female only, mixed); and weather (no sun, partial sun, sunny). As multiple females were observed from the same herd, herd identity was treated as a random effect. We framed an a priori candidate set of 27 models (Appendix S5) including both the null and the global model.

## 3 RESULTS

### 3.1 Habitat‐use pattern

Out of a total of 672 data points across three months, blackbuck indirect signs were recorded in 99 sampling trials. Of these, 97 were from inside the CA and only two from outside. Indirect signs were present in 44 of 224 unique sampling points. Also, among these 44 sampling points, some were highly populated with signs indicating intensive habitat use in some of the grids.

Even inside the core area, among the four types of habitats present, no blackbuck indirect sign was obtained from sample points that lay in dense forests. Signs were mostly concentrated in grasslands (67% of 114 trials) and *Bombax* forest (70% of 27 trials), and only one trial (*n* = 30) in croplands inside the CA showed blackbuck signs (Appendix S6).

**FIGURE 3:**
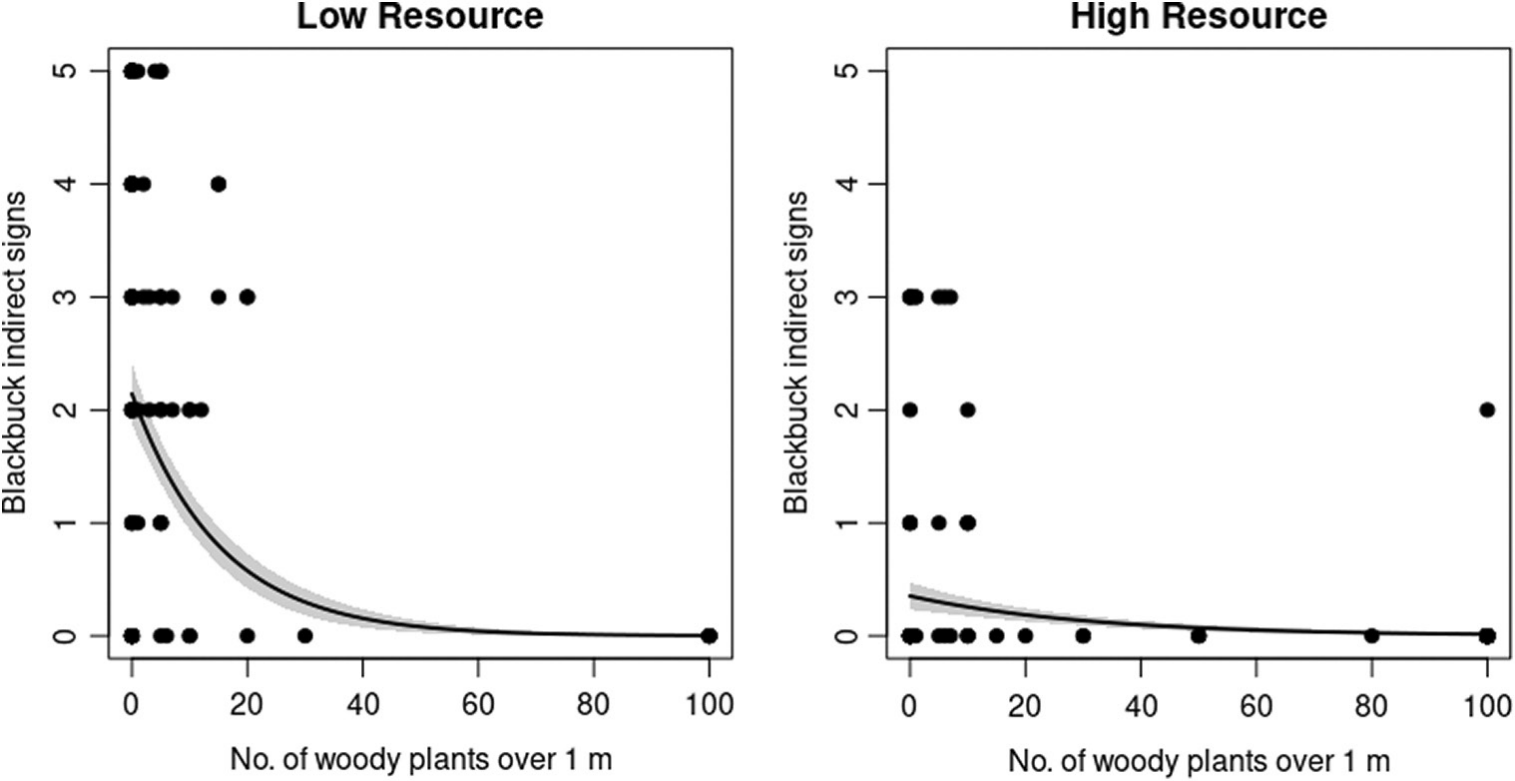
The relationship between blackbuck habitat‐use and habitat openness (number of plants over 1 m, a risk factor) at different levels of resource abundance. Resource abundance was modeled as a continuous variable. Here, it has been categorized to visualize the statistical interaction between habitat openness and resource abundance. The lines represent model predictions. The bands represent 1 SE.

**FIGURE 4:**
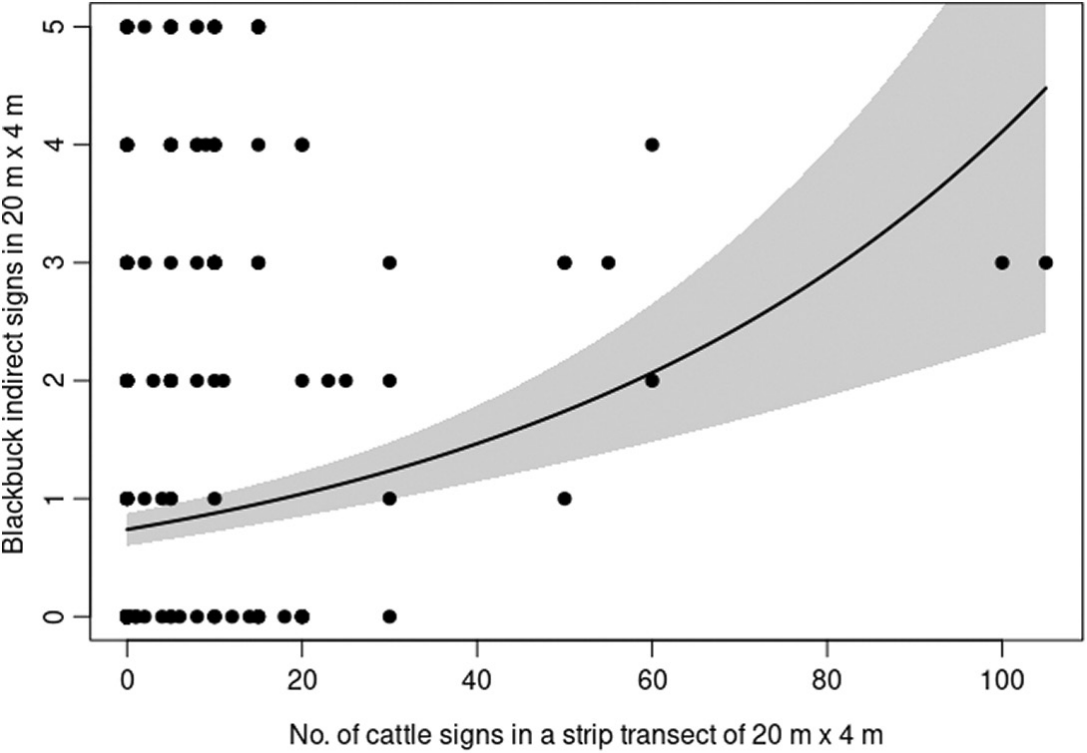
The correlation between blackbuck habitat‐use and livestock signs inside the core area. The line represents model predictions. The band represents 1 SE.

The habitat use analysis indicated that both risk factors and resources influence blackbuck habitat use (Appendix S7). Blackbuck habitat use varied with the abundance of woody plants over 1 m (model averaged weight = 1), resource abundance (model averaged weight = 1) (Figure 3), and their interaction (model averaged weight = 1). The model averaged coefficients and 95% confidence intervals indicated that where resource abundance was low, habitat use was negatively related to the frequency of tall woody plants. This relationship weakened as resource abundance increased (Table 1). Blackbuck habitat use was positively correlated with cattle sign abundance (model averaged weight = 0.99) (Figure 4). There was only weak support for the effect of an interaction between distance and resource on blackbuck habitat use (model averaged weight = 0.52) (Table 1). When resource abundance was low, habitat use was similar at different distances from the center of the core area. However, in areas with higher levels of resource abundance, habitat use was greatest in the core center and decreased toward the periphery (Table 1).

**TABLE 1:** Model averaged β‐coefficients, 95% confidence limits, and weights associated with different predictors in the analysis of blackbuck habitat use (through indirect signs). The model set comprised 24 models fitted using GLMMs.

	B Estimate	95% Lower CL	95% Upper CL	Weights
**Intercept**	**−3.78**	**−5.80**	**−1.77**	
**Pover**	**−6.99**	**−10.97**	**−3.02**	**1.00**
Resource	0.005	−0.71	0.71	1.00
**Cattle**	**0.22**	**0.09**	**0.35**	**0.99**
Distance	−0.02	−0.41	0.37	0.78
**Pover:Resource**	**2.47**	**1.34**	**3.60**	**1.00**
**Distance:Resource**	**−0.48**	**−0.99**	**−0.03**	**0.52**

*Note*: CL—confidence limit; Pover—total number of plants over 1 m in height in 10 m radius from sample point; Resource—averaged product of plant height and percentage coverage at 1 m and 20 m; cattle—total cattle sign (dung piles and hoof marks) in 20 m × 4 m area of the transect; Distance—linear distance of each sampling point from the center of the core. Terms in bold indicate 95% confidence intervals that do not include zero.

### 3.2 Behavior assessment

We scanned animals for 89 h in total. As each hour (independent observation session) had seven observations, we had altogether 623 behavior scans of groups of animals, which comprised 12,811 individual instantaneous samples. The mean group size during 89 h of observation was 20.56 ± 18.61 (mean ± SD).

As the youngest animals in the area were already between 8 and 9 months old (last fawning month was 9 months before the field season started) we could not separate fawn from male and female during observation and a few individuals were unidentified. We did not include unidentified individuals in analyses. So, the analyses included adult males (males with three or more curls in the horn), immature males, and females. Animals observed in more than two‐thirds of the scans were females.

### 3.3 Foraging

**FIGURE 5:**
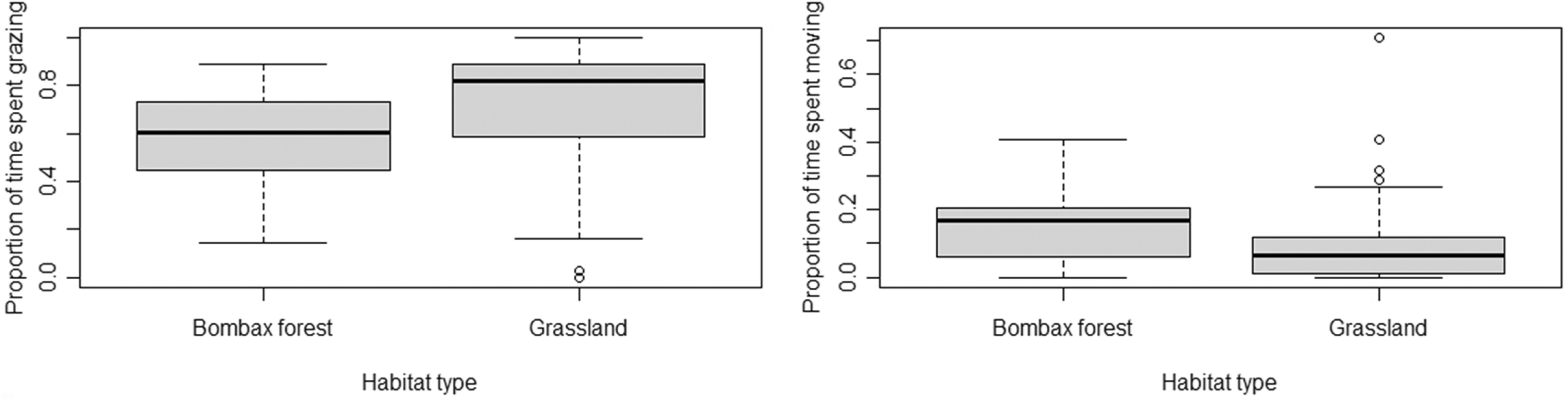
Proportion of time spent foraging (left), moving (right) by blackbuck in two different habitat types. The bold lines represent the median; the box represents the interquartile range, and the whiskers represent the data extremes.

Foraging was the most prominent activity observed during scans, forming two‐thirds of the total number of scans. Our model that incorporated only habitat type best‐predicted foraging behavior in blackbuck (Appendix S8; model averaged weight of habitat type = 0.69). Although animals were observed feeding on similar grass species in both habitat types, they spent a greater proportion of their time foraging in grassland than in forest patch. However, this relationship showed some uncertainty (Figure 5). Other predictors like time of the day (model averaged weight = 0.45), group type (model averaged weight = 0.31), weather (model averaged weight = 0.37), location (model averaged weight = 0.21), and mean group size (model averaged weight = 0.21) did not have much influence on foraging in blackbuck (Appendix S9).

### 3.4 Moving

Moving was the second most prominent activity observed during scans. Our model that incorporated habitat type, mean group size and time of the day was the best predictor of moving behavior in blackbuck (Appendix S10). The proportion of time spent moving varied most with habitat type (model averaged weight = 0.93). Animals spent less time moving in grassland than in forest patches (Figure 5). Other predictors like time of the day (model averaged weight = 0.57), mean group size (model averaged weight = 0.54), location (model averaged weight = 0.19), weather (model averaged weight = 0.05), and group type (model averaged weight = 0.04) did not have much influence on moving behavior in blackbuck (Appendix S11).

### 3.5 Vigilance behavior

In total, we observed 186 female blackbuck for 1 min each. These females were not marked. While care was taken to avoid sampling the same individual during a sampling session (see Methods), it is possible that there were repeated observations of the same individual from different sampling occasions. The mean vigilance frequency was 1.24 ± 1.16 (mean ± SD; range = 0–5).

**FIGURE 6:**
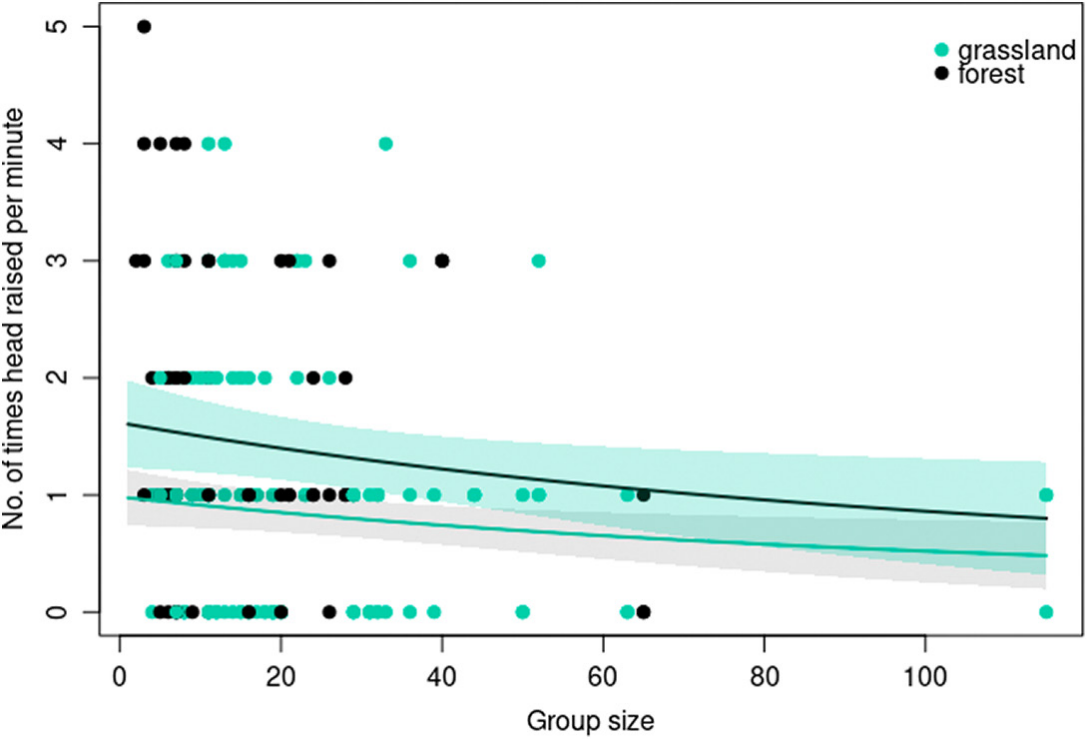
Variation in vigilance frequency in relation to group size and in two different habitat types. Lines are GLMM model predictions using model averaged coefficients. The bands represent 1 SE.

Our model that included group size, habitat type, and weather was the best predictor of vigilance by blackbuck (Appendix S12). Vigilance in blackbuck varied the most with habitat type (model averaged weight = 0.93). Females were less vigilant in grasslands than in forest patches. Weather (model averaged weight = 0.84) and group size (model averaged weight = 0.71) were also observed to influence vigilance behavior substantially (Figure 6). Mostly, females were less vigilant on a sunny day than on a completely foggy or partial sunny day whereas vigilance frequency between completely foggy or partial sunny did not differ much. Vigilance decreased as group size increased. There was very little support for the influence of group type (model averaged weight = 0.35) and location (model averaged weight = 0.34) on vigilance behavior (Appendix S13).

## 4 DISCUSSION

### 4.1 Habitat‐use pattern

Our findings indicated that habitat use by blackbuck in a human‐dominated landscape was strongly affected by risk factors, related to both habitat and human activities. First, blackbuck signs in areas outside the core area, in the Community Development Zone (CDZ), were negligible. This indicates a strong negative impact of high levels of human activity on blackbuck habitat use. Blackbuck signs were not detected even in areas of high resource abundance within the CDZ, namely crop fields with wheat, lentils, and maize. In earlier work based on a social survey conducted at the same site, people have identified these crops as those most used by blackbuck and have reported that these crops were damaged the most by blackbuck (Kuwar, 2015). Future work at this site should investigate the concordance between evidence for damage from social surveys and from field measurements; and between the perception of damage and actual damage. The lack of blackbuck use of the CDZ suggests that blackbuck prioritize risk factors over resources when making habitat‐use decisions in areas with a high degree of human activities. While certain species appear to adapt well to highly human‐modified environments (e.g., reviewed in Lowry et al., 2013), our results are similar to those from several studies that report adverse impacts of human activities on wild ungulate habitat use. A recent study by Costa et al. (2021) shows that both single‐season and multiseason farmlands constrict the habitat use of wild ungulates. Similarly, another recent study has found mule deer considerably reducing habitat use in areas with human‐induced noise (Kleist et al., 2021). These studies, like most previous work, are from landscapes with low densities of humans. Our study contributes to this literature by providing information from a heavily human‐dominated landscape.

Interestingly, our study also indicates that blackbuck are able to persist in human‐dominated landscapes in areas with low levels of anthropogenic factors. Within the core area, blackbuck signs were abundant even though there is some human activity, although at much lower levels when compared with the CDZ. In the CDZ, there were hardly any areas that could not be categorized as either built‐up areas or crop fields, whereas such land uses were limited inside the CA (KrCA, 2017).

Furthermore, supporting our prediction, resource availability and risk factors together best‐explained habitat use by blackbuck within the core area. Risk associated with habitat, specifically how close the habitat was (indexed by the abundance of tall woody plants in the area), was consistently negatively correlated with habitat use by blackbuck. Blackbuck habitat use was the greatest in open habitats without tall woody plants and decreased sharply as the habitat became more closed. This relationship weakened as resource availability increased. Blackbuck habitat use did not clearly increase with resource availability. This is likely because blackbuck prefer short grass habitats and are less likely to use tall grass areas. Jhala (1991) found that blackbuck mostly use grassland with short grass with a height of less than 50 cm and they avoided areas with tall grasses. Tall grass can obstruct visibility that is directly associated with predation risk. Furthermore, tall grass is mostly matured with coarse edges and is thus, of comparatively low nutritional quality. Blackbuck selectively feed on more nutritious grass parts and feed less on coarse forage (Jhala, 1997). Krishna et al. (2016) also found that blackbuck prefer relatively short grass to tall grass areas. This study, which explored blackbuck habitat use in a human‐dominated landscape in India, also reported an interaction between resource and risk, as in our study.

Another line of evidence that blackbuck can persist alongside low human activity is provided by our results that blackbuck regularly use areas with livestock presence. This finding has important implications considering the situation where cattle grazing is strictly prohibited in most blackbuck ranging protected areas in Nepal and India. This finding is in line with earlier work that has shown that livestock foraging can positively influence wildlife (Schieltz & Rubenstein, 2016). Many recent reviews have also suggested that light to moderate livestock foraging in grasslands is more beneficial in terms of vegetation productivity and quality than a complete lack of foraging (Holechek et al., 2006). This might explain why blackbuck are attracted to those patches of grassland that are used by livestock. Alternatively, the correlation between blackbuck habitat use and livestock signs might be an outcome of these two species independently selecting similar habitat conditions. However, detailed studies are needed to elucidate whether the interaction between the two species is characterized by antagonisms, competition for resources, or potentially commensalism.

### 4.2 Behavioral variation

Investigating the behavior of animals in different habitats can provide insights into how animals respond to changing risk and resource factors. We examined behavioral responses likely to be affected by risk. In large herbivores, group‐living and vigilance are responses to reduce the risk from threats such as predation (Isvaran, 2007). Other behaviors, such as foraging and moving, may also provide insights into the costs and benefits associated with different types of habitats. As predicted, vigilance behavior varied with herd size and habitat structure. Animals in larger herds were less vigilant. Such a relationship between vigilance and group size has been shown in many species (Isvaran, 2007) and is thought to arise from the benefits of shared vigilance in larger groups. This reduction allows animals to allocate more time to other key activities, such as foraging (Roberts, 1996), and to increase daily activity levels (Ramirez et al., 2021).

Blackbuck were less vigilant in grassland than in adjoining *Bombax* forest, which had scattered trees with shrubby undergrowth that measured approximately one m in height. Vigilance behavior is expected to differ with the level of predation risk. As understorey cover obstructs the detection of predators, the vigilance rate is expected to increase in such a habitat when compared with open grassland (Blank, 2018; Ebensperger & Hurtado, 2005). Isvaran (2007) also found that habitat structure played a role in deciding trade‐offs between foraging and vigilance in blackbuck. Blackbuck, on average, spent a larger proportion of their time foraging in grasslands than in adjoining *Bombax* forest patches. This difference is likely related to both differences in forage availability and in risk. More undergrowth in forest patches likely reduces grass availability and increases obstruction to vision resulting in increased risk. Like foraging, moving behavior in blackbuck was also best explained by habitat type. However, in contrast to foraging, blackbuck moved less in grassland and more in *Bombax* forest. This difference might also be explained by the same factors—obstruction to vision and reduced grass patches due to undergrowth. With the increase in risk and patchiness of grass in *Bombax* forest, blackbuck might move more in this habitat than in grassland.

This study has implications for the long‐term persistence of blackbuck in the study site, an area of high conservation priority. First, blackbuck appear to strictly avoid areas largely consisting of agricultural fields and built‐up areas and without any grassland or forest patches. At the study site, blackbuck primarily use the core area, which is a relatively small area comprising of grassland and *Bombax* forest within the larger landscape. If direct and indirect human activity increases in the core area too, our study indicates that the long‐term persistence of blackbuck in the study area will be negatively affected. Second, KrCA has a history of people grazing their livestock even inside the core area. It continues to be a major demand of people living inside and around the CA. A complete ban on livestock grazing is likely to receive opposition from local communities and may thus, pose a challenge to conservation at the study site. As our work shows, livestock foraging does not appear to negatively affect the use of an area by blackbuck. Therefore, management strategies can be explored that permit livestock foraging in selected parts of the core area.

In conclusion, our findings indicate that both ecological and anthropogenic factors influence habitat use by blackbuck in this human‐dominated landscape. Blackbuck appear to be sensitive to risk associated with both natural and anthropogenic factors. Our work suggests that wild herbivores may be able to persist in landscapes with high human densities so long as there are refuges where levels of human activity are relatively low.

This work is one of the first to examine the impacts of direct and indirect human presence on the ecology and behavior of a comparatively large and threatened wild mammal in a highly human‐dominated landscape. It paves the way for future studies to investigate the processes by which anthropogenic factors affect animal ecology and behavior in such landscapes. For example, in‐depth studies of direct and indirect interactions between livestock, wild herbivores, and their forage can help to uncover causal mechanisms by which livestock impact wild herbivores. Similarly, studies that investigate how anthropogenic factors modify the “landscape of fear” experienced by wild herbivores can reveal their direct (e.g., mortality from human‐related factors) and indirect (e.g., reduced body condition through reduced use of risky habitats with high food abundance) effects on these populations. Finally, the continued monitoring of animal populations, their ecological conditions, and their interactions with humans in such landscapes are crucial to establish whether the long‐term persistence of such large wild herbivores is possible in highly human‐dominated landscapes.

## AUTHOR CONTRIBUTIONS

**Rohit Raj Jha:** Conceptualization (equal); data curation (lead); formal analysis (lead); methodology (equal); writing – original draft (lead). **Kavita Isvaran:** Conceptualization (equal); formal analysis (supporting); methodology (equal); supervision (lead); validation (equal); writing – original draft (supporting).

## CONFLICT OF INTEREST

None.

## Supporting information


Appendix S1–S13

## Data Availability

All the relevant data used in this study are archived in Dryad and can be accessed at https://doi.org/10.5061/dryad.jq2bvq89k.
